# A Large Tricuspid Subvalvular Apparatus Infective Endocarditis Undetected by Transthoracic Echocardiography

**DOI:** 10.7759/cureus.58477

**Published:** 2024-04-17

**Authors:** Johnathon J Rast, Zoheb Sulaiman, Kayla Shahbazian, Ashley Huggett

**Affiliations:** 1 Internal Medicine, WellStar MCG Health, Augusta, USA; 2 Infectious Disease, WellStar MCG Health, Augusta, USA; 3 Cardiology, WellStar MCG Health, Augusta, USA

**Keywords:** septic emboli, transthoracic and transesophageal echocardiography, tricuspid valve repair, intravenous drug use (ivdu), right sided infective endocarditis

## Abstract

A 39-year-old male with a history of intravenous drug use (IVDU) and no significant cardiovascular disease was admitted to the ICU for management of septic shock and acute hypoxic respiratory failure secondary to septic pulmonary emboli. Due to a high clinical suspicion for right-sided infective endocarditis (IE), he received a transthoracic echocardiogram (TTE), which did not reveal any vegetations. However, a transesophageal echocardiogram (TEE) was subsequently performed; this showed a large 2.4 cm vegetation in the septal aspect of the tricuspid valve (TV) subvalvular apparatus. He urgently underwent surgical removal of the vegetation and repair of the TV. Postoperatively, he clinically recovered with appropriate antibiotic therapy.

TEE is the ideal imaging modality in evaluation for IE, but a minimally invasive TTE is often performed first. This case highlights a highly unusual anatomic location of IE, which harbored a large vegetation undetected by TTE. In patients without cardiac devices or non-native valves, an urgent TEE remains diagnostically essential if there is a high clinical suspicion for right-sided IE, even if a TTE shows no evidence of IE.

## Introduction

*Staphylococcus aureus* bacteremia is a well-known complication of intravenous drug use (IVDU). Non-sterile needles penetrate through unclean skin, which facilitates translocation of bacterial skin flora, including *Staphylococcus aureus*, into the bloodstream. As bacteria enter the venous system, they first encounter the right side of the heart. Their biofilm can facilitate adherence to native cardiac valves, prosthetic valves, and other intracardiac devices if present. The tricuspid valve (TV) is the most commonly affected structure in right-sided infective endocarditis (IE) [1]. Although right-sided IE carries a lower mortality and lower population frequency than left-sided IE, right-sided IE remains a feared complication associated with IVDU [1]. Clinicians must maintain a high clinical index of suspicion when managing patients who inject IV drugs that present with unexplained fevers, sepsis, or evidence of septic embolization from right-sided cardiac structures. Echocardiography is the imaging tool of choice for initial evaluation [1]. Transthoracic echocardiography (TTE) is typically performed before transesophageal echocardiography (TEE) due to the more rapid and less invasive nature of the modality. In patients without prosthetic valves or intracardiac devices, TTE appears to have equivalent sensitivity as TEE for diagnosing right-sided IE [2-5]. We present a case of right-sided IE associated with IVDU consisting of a large vegetation seated in the tricuspid subvalvular apparatus. This large vegetation, measuring 2.4 cm in length, was initially undetected on TTE. Only subsequent imaging with TEE identified this vegetation, contributing to a delay in appropriate management of this critical illness.

## Case presentation

A 39-year-old male with a past medical history significant for active intravenous drug abuse, lumbar spondylolisthesis requiring posterior lumbar interbody fusion, major depressive disorder, and non-insulin dependent type 2 diabetes mellitus presented to the emergency department for a constellation of subjective fevers, chills, chest pain, and worsening dyspnea on exertion for five days. His symptoms began shortly after a physical altercation, which led to him being stabbed in his left leg. Vitals signs on admission included a blood pressure of 80/40 mmHg, tachycardia to 118 bpm, tachypnea to 21 breaths/minute, oxygen saturation of 96% on room air, and a temperature of 36.7°C. Cutaneous signs of IVDU were not well seen, but a non-raised erythematous lesion was visualized on his left anterior shin.

An initial CT head and cervical spine with IV contrast did not find any acute pathology. A CT chest angiography revealed multiple peripheral thick-walled cavitary pulmonary lesions, which were suspicious for septic pulmonary emboli. Initial laboratory studies are depicted in Table 1. His electrocardiogram showed sinus tachycardia without any signs of myocardial infarction or ischemia. Blood cultures returned positive for methicillin-sensitive *Staphylococcus aureus* (MSSA) and *Streptococcus mitis* via blood culture identification panel. He initially responded to aggressive IV fluid resuscitation, but his septic shock progressed, resulting in transfer to the ICU for vasopressor support with norepinephrine infusions.

**Table 1:** Initial Laboratory Results BUN: Blood urea nitrogen; CRP: C-reactive protein; ESR: Erythrocyte sedimentation rate

Laboratory Study	Laboratory Value	Normal Value Range
White Blood Cells	13,100/mm^3^	4,500 - 11,000/mm^3^
Hemoglobin	13.0 g/dL	14 - 18 g/dL
Platelets	176,000/mm^3^	150 - 400/mm^3^
Sodium	131 mEq/L	132 - 146 mEq/L
Chloride	96 mEq/L	99 - 109 mEq/L
BUN	19 mg/dL	9 - 23 mg/dL
Creatinine	1.3 mg/dL	0.6 - 1.6 mg/dL
Glucose	97 mg/dL	74 - 106 mg/dL
Lactic Acid	1.81 mmol/L	0.5 - 2.2 mmol/L
ESR	91 mm/hr	< 21 mm/hr
CRP	27.557 mg/dL	< 0.500 mg/dL
Urine Opiates	Positive	Negative

**Figure 1:**
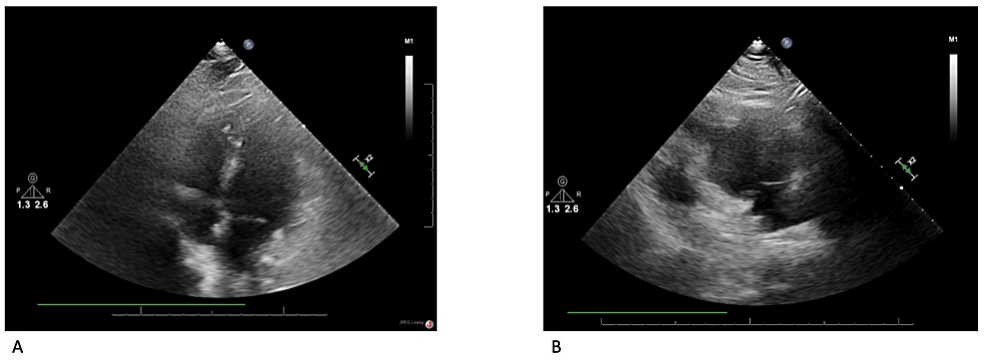
TTE Figure 1A depicts the TTE Apical 4-Chamber view. Figure 1B depicts the RV inflow view. The TV septal leaflet and the RV septum can be visualized in each view without any clear evidence of vegetation on the TV or in the RV. TTE: Transthoracic echocardiogram; RV: Right ventricle; TV: Tricuspid valve

He was started on empiric antibiotics with vancomycin and then de-escalated to cefazolin to target his MSSA bacteremia. A TTE was performed due to high clinical suspicion for right-sided IE, which revealed normal right and left ventricular systolic function, mild tricuspid regurgitation, and no vegetations visualized in the right side of the heart (Figure 1). CT lumbar spine with IV contrast revealed a small hypoechoic density within the right paraspinal musculature (1.4 cm x 1.7 cm) without organized fluid collection to suggest abscess or osteomyelitis. His encephalopathy worsened leading to emergent endotracheal intubation. Given his worsening encephalopathy, there was concern he could have septic cerebral emboli so cefazolin was discontinued in favor of nafcillin for better central nervous system penetration. A repeat CT head revealed no evidence of septic cerebral emboli.

**Figure 2:**
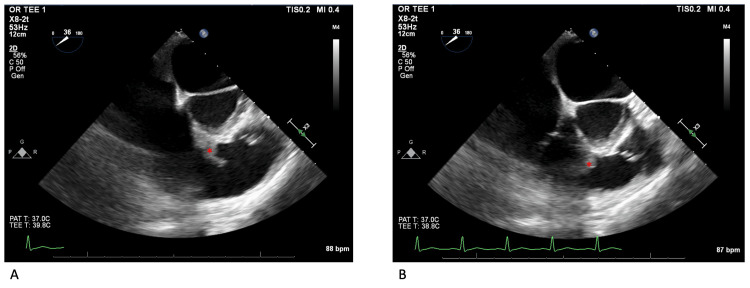
Transesophageal Echocardiography Figures 2A-B depict the TEE midesophageal RV inflow/outflow view. Figure 2A shows the vegetation extended towards the RV outflow tract in the septal aspect of the TV subvalvular apparatus. Figure 2B displays further extension of the vegetation into the RV outflow tract, which was concerning for a dynamic partial RV outflow tract obstruction. The red asterisk marks indicate the infectious vegetations. TEE: Transesophageal echocardiogram; RV: Right ventricle; TV: Tricuspid valve

A TEE was then performed, which was negative for vegetation of the tricuspid or pulmonic valve leaflets. However, a large pedunculated mobile mass, measuring 2.4 cm, was visualized with attachment to the right ventricular basal septal wall and the septal tricuspid subvalvular apparatus (Figure 2). The patient underwent cardiac surgery the following day. He was placed on cardiac bypass, and the intracardiac space was accessed via an incision in the right atrium. A small abscess was visualized on the septal aspect of the tricuspid subvalvular apparatus in addition to the large vegetation in the subvalvular apparatus inferior to the septal leaflet of the TV. The vegetation was removed and the small septal subvalvular abscess was debrided. Intraoperative tissue from the vegetation and abscess confirmed IE by pathological report. The patient's postoperative period was without significant complications, including completion of IV nafcillin for six weeks after valve surgery.

## Discussion

IE most commonly affects the left-sided heart structures with the right side of the heart involved in only 5-10% of cases [6]. However, in patients who inject IV drugs, right-sided IE appears to be more common than left-sided IE [7]. Additionally, about 90% of right-sided IE cases involve patients with a history of IVDU [6]. TEE is the gold standard imaging modality for diagnosing IE [1]. However, TTE and TEE appear to have similar sensitivity for diagnosing most cases of right-sided IE in the absence of intracardiac devices and non-native cardiac valves [2-5]. Right-sided cardiac structures are seated anterior in the mediastinum, and thus closer to the TTE probe, which may explain the similar sensitivity between the two imaging modalities. The TV leaflets are the most common location of attachment for right-sided IE [2,7]. It is possible that TTE may have significantly inferior sensitivity for detecting right-sided IE seated in atypical locations compared to TEE, but no large studies exist thus far to evaluate this uncertainty. 

This patient demonstrates an atypical location of right-sided IE, which was undetected by TTE. Despite a persistently high clinical suspicion, the urgency of TEE was reduced from the TTE findings. Thankfully, TEE delay did not appear to negatively impact the patient’s outcome. Although several factors are associated with delayed diagnosis and treatment of IE, delayed IE diagnosis is associated with significantly higher mortality rates [8-10]. Epidemiologic data suggest that right-sided IE associated with IVDU is rising in frequency, thus increasing the importance of a high clinical index of suspicion for providers caring for patients at risk [11]. Earlier diagnosis leads to prompt treatment; however, optimal management of right-sided IE remains unclear, in part due to ethical implications. As previously mentioned, right-sided IE is strongly associated with IVDU, and surgical implantation of a replacement heart valve (representing a future infectious nidus) in a patient strongly predisposed to re-infection remains controversial. A careful multidisciplinary approach is required to weigh several factors, including likelihood of successful treatment with antibiotics alone, the degree of structural damage already incurred, perioperative mortality risk, the likelihood of the patient abstaining from IVDU in the future, and the patient’s personal wishes. Whether surgery is performed or not, antimicrobial therapy is always indicated in management of IE. Antimicrobial selection and duration is dependent on the underlying organism and antimicrobial susceptibility [1]. 

An estimated 85% of cases of MSSA native valve IE associated with IVDU can be cured with recommended antibiotic regimens [1]. Typically, an effective regimen includes a beta-lactam with or without gentamicin, with duration ranging between four to six weeks, largely depending on whether the infection is considered complicated [1]. Large vegetations, septic embolization, and continually deteriorating clinical status may reduce the likelihood of effective treatment with antibiotics alone, warranting evaluation for surgical management [1].

## Conclusions

This case highlights an unusual location of right-sided IE, which may be a location of poor diagnostic sensitivity for TTE. This case exemplifies that an initial TTE cannot always rule-out right-sided IE even when large vegetations are present. Furthermore, this emphasizes the importance of urgent further evaluation with TEE, even with an unremarkable TTE, in patients with high clinical suspicion for right-sided IE.
